# Identification of novel α7 nicotinic receptor ligands by in silico screening against the crystal structure of a chimeric α7 receptor ligand binding domain

**DOI:** 10.1016/j.bmc.2012.06.054

**Published:** 2012-10-01

**Authors:** Atilla Akdemir, Ewald Edink, Andrew J. Thompson, Sarah C.R. Lummis, Albert J. Kooistra, Chris de Graaf, Iwan J.P. de Esch

**Affiliations:** a Division of Pharmacology, Faculty of Pharmacy, Bezmialem Vakif University, Istanbul, Turkey; b Leiden/Amsterdam Center of Drug Research (LACDR), Division of Medicinal Chemistry, Amsterdam Institute for Molecules, Medicines and Systems (AIMMS), VU University Amsterdam, The Netherlands; c Department of Biochemistry, University of Cambridge, Cambridge, UK

**Keywords:** α7 Receptor, nAChR, AChBP, Virtual screening, In silico screening, Docking, Cys-loop

## Abstract

A hierarchical in silico screening procedure using the crystal structure of an agonist bound chimeric α7/Ls-AChBP protein was successfully applied to both proprietary and commercial databases containing drug-like molecules. An overall hit rate of 26% (p*K*_i_ ⩾5.0) was obtained, with an even better hit rate of 35% for the commercial compound collection. Structurally novel and diverse ligands were identified. Binding studies with [^3^H]epibatidine on chimeric α7/5-HT_3_ receptors yielded submicromolar inhibition constants for identified hits. Compared to a previous screening procedure that utilized the wild type Ls-AChBP crystal structure, the current study shows that the recently obtained α7/Ls-AChBP chimeric protein crystal structure is a better template for the identification of novel α7 receptor ligands.

## Graphical abstract

**Figure:**
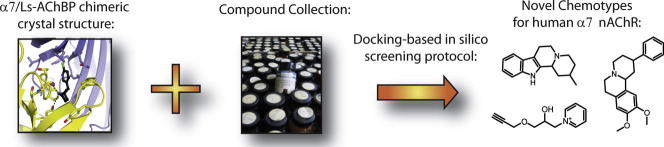

## 1 Introduction

Nicotinic acetylcholine receptors (nAChRs) are members of the Cys-loop family of ligand-gated ion channels (LGICs).^1–3^ This family also includes receptors for serotonin (5-HT_3_R), γ-aminobutyric acid (GABA_A_R) and glycine (GlyR).^1–3^ The Cys-loop receptors all share a common architecture of five subunits that combine to form an agonist-responsive receptor with an integral ion channel (with approximately fivefold symmetry) that spans the cell membrane. Within each receptor type there are several subunits and different combinations of these subunits form receptor subtypes with different pharmacological characteristics.^1–4^ The human nAChR subunits include α1–α7, α9, α10, β1–β4, δ, ε and γ subunits. In principle, there are many possible combinations, but two nAChR subtypes are most commonly seen in the central nervous system (CNS), namely α4β2 and α7 receptors.^5^ These are mainly found on neuronal cells in the CNS where they are considered to be important drug targets in CNS disorders such as Alzheimer’s disease, Parkinson’s disease, schizophrenia and some forms of epilepsy.^1–4^ These nAChRs are also involved in the physiology of anxiety, memory, cognition, pain and addiction.^1–4^ The human α7 receptor is also found on non-excitable cells in the periphery where it is believed to be a pharmacological target for inflammation and some forms of cancer.^6,7^

Insight into the structure of the nicotinic receptor ligand-binding domain (LBD) has been derived from high resolution crystal structures of acetylcholine-binding proteins (AChBPs).^8–11^ AChBPs have been recognized as water-soluble homologs of the LBD of LGICs and have been obtained from different snail species such as *Lymnaea stagnalis* (Ls-AChBP),^8,9^ A*plysia californica* (Ac-AChBP)^11^ and *Bulinus truncatus* (Bt-AChBP).^10^ AChBPs show similarity in sequence identity (∼20–24% between AChBPs and nAChRs) and structure (similar size and architecture, pentameric assembly) to the LBD of nicotinic receptors.^8–11^ In particular, aromatic residues that form the binding pocket are conserved between AChBPs and nAChRs, and AChBPs bind several reference nicotinic receptor ligands such as nicotine, epibatidine and lobeline. As AChBPs are extracellular and water soluble proteins that can be obtained in relatively high yields, several have been crystallized in complex with selective and high affinity nicotinic receptor ligands, for example, Ls-AChBP in complex with nicotine and Ac-AChBP in complex with epibatidine.^11,12^ The availability of these AChBP crystal structures has significantly improved our understanding on the overall molecular structure of the nicotinic receptor LBD and its molecular interactions with nicotinic ligands. Nevertheless, due to its moderate overall sequence identity (24% with the human α7 receptor LBD), AChBP cannot be considered an exact water-soluble mimic of the nicotinic receptor LBD. Recent work has shown that structure–activity relationships (SAR) that were identified for AChBP do not always correlate with SAR for α7 or the α4β2 nAChRs.^13,14^ Deviation in amino acid composition between AChBP and nicotinic receptors, particularly in the complementary face of the binding site, are likely causes for the observed SAR differences.

Despite these structural differences, AChBP crystal structures have been successfully used in in silico (virtual) screening procedures by our research group^15,16^ and others,^17,18^ and novel ligands with affinity for AChBPs and nAChRs have been obtained. In our first in silico screen, we used crystal structures of Ls-AChBP in complex with nicotine (PDB: 1UW6), HEPES (PDB: 1UX2) and carbamylcholine (PDB: 1UV2).^15^ Using identical protocols and screening libraries, the crystal structures in complex with nicotine and HEPES performed considerably better in the identification of novel AChBP and α7 receptor ligands, compared to the Ls-AChBP crystal structure in complex with carbamylcholine. These findings show that small structural differences between crystal structures of a specific protein can have profound effects on the outcome of in silico screening campaigns.

A recently refined in silico screening procedure (using the Ls-AChBP crystal structure in complex with nicotine) was able to identify novel and diverse scaffolds for the target protein AChBP,^16^ but it was less successful in identifying ligands for its therapeutically relevant structural homolog, the α7 nicotinic receptor. This clearly indicates some of the limitations of using AChBP as a template for finding nicotinic receptor ligands.

Very recently, a crystal structure of a chimeric α7/Ls-AChBP protein in complex with the agonist epibatidine has been obtained by Li and co-workers.^19^ This protein shares 64% sequence identity with the human α7 extracellular LBD and residues lining the binding pocket are completely derived from the α7 receptor. Having detailed structural information of the binding pocket of a very close homolog to the human α7 receptor, we applied our in silico screening procedure on this agonist-bound crystal structure with the aim of identifying novel chemotypes for the α7 nicotinic receptor.

Our hierarchical in silico screening procedure was successfully applied on a commercial compound collection to identify novel α7 receptor ligands with a good hit rate of 35%. As such, our efforts have resulted in identification of a set of structurally diverse chemical starting points that can be used for further optimization towards novel high affinity α7 nicotinic receptor ligands. When comparing to our previous Ls-AChBP-based in silico screening exercise, the current study shows that the recently obtained α7/Ls-AChBP chimeric protein crystal structure is a better template for identification of novel α7 receptor ligands than the previously used Ls-AChBP crystal structure.

## 2 Results

### 2.1 Comparing the binding pockets of the chimeric α7/Ls-AChBP protein and Ls-AChBP protein

**Figure 1:**
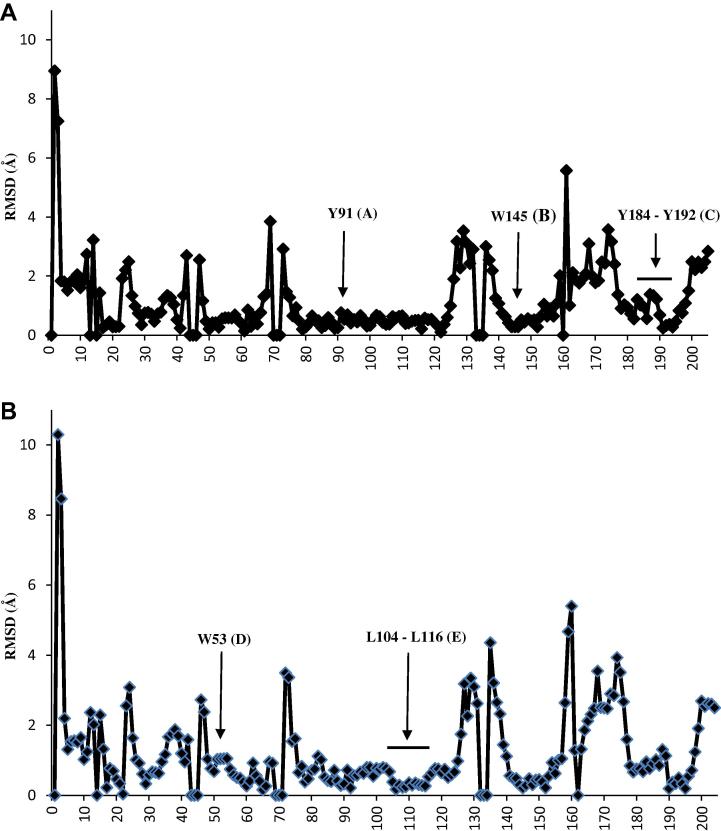
RMSD values per residue (α7/Ls-AChBP chimera numbering) for the principal (A) and complementary (B) subunits. The binding pocket residues Y91 (loop A), W145 (loop B) and Y184–Y192 (loop C) of the principal side and residues W53 (loop D) and L104–L116 (loop E) of the complementary side all have RMSD values lower than 2 Å.

**Figure 2:**
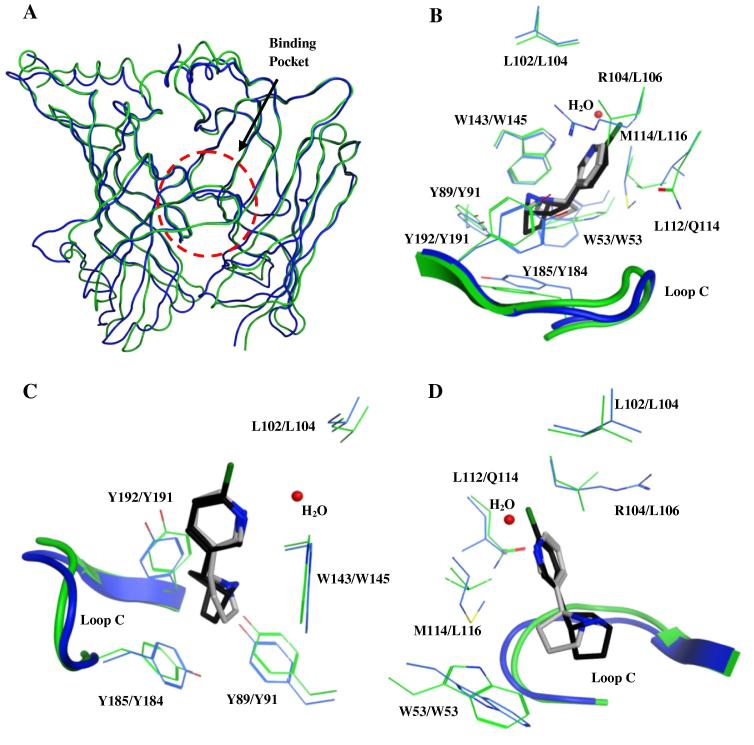
Structural comparison between Ls-AChBP (blue) and the α7/Ls-AChBP chimeric protein (green). (A) In general, the backbone fold between the two proteins is similar, especially close to the binding pocket, which is located within the circle (red dashed circle). (B) The residues aligning the binding pocket in general superpose well. However, there are differences in amino acid composition and loop conformation. (C) For the principal side, the aromatic residues are conserved and loop C has a similar fold in both proteins with only minor differences in the tip of loop C. (D) The residues of the complementary side are not conserved between both proteins, with the exception of W53 (both Ls-AChBP and α7/Ls-AChBP numbering) and L102 (Ls-AChBP numbering). However, differences in the side chain orientation exist for W53. Carbon atoms of Ls-AChBP and the α7/Ls-AChBP chimeric protein are depicted in blue and green, respectively. Ligand carbon atoms are depicted in grey for nicotine and black for epibatidine. The bridging water molecule of the nicotine-bound Ls-AChBP structure is depicted in red. L102 and L104 are shown in C and D for clarity. Residue numbering is depicted as ‘Ls-AChBP residue number’/‘chimeric protein residue number’.

We compared the subunits A and B of both the chimeric α7/Ls-AChBP protein (PDB: 3SQ6; residues lining the binding pocket are completely derived from the α7 receptor) and Ls-AChBP (PDB: 1UW6), by means of a structural superposition using their Cα-atoms. The Cα-atoms of both proteins superpose well (principal face: RMSD = 1.7 Å for 204 residues; complementary face: RMSD = 1.8 Å for 204 residues) and the RMSD per residue (Cα-atoms) of the principal and complementary sides are depicted in Figure 1A and B, respectively. Most of the residues have low RMSD values (⩽1 Å), but several RMSD values are above 2 Å. These residues with higher RMSD values for their Cα-atoms (especially RMSD ⩾2 Å), indicating a difference in the position of the backbone atoms, are all located outside the binding pocket (Figs. 1A, B and 2A). Nevertheless, the superposition also reveals considerable differences in residue composition and side chain orientation between the binding sites of both proteins (e.g., W53 and the tip of loop C; Fig. 2B–D). As a result, some notable differences in possible ligand–protein interactions between the two structures can be observed. For example, L112 in wild type Ls-AChBP is replaced by a substantially more polar Q114 in the chimeric α7/Ls-AChBP protein and may provide an opportunity to obtain specific polar interactions with the α7 binding site. The specific rotameric configuration of W53 in the chimeric α7/Ls-AChBP enables another possibility for formation of polar interactions with the binding site. In contrast to the wild type Ls-AChBP structure, W53’s indole nitrogen atom is pointing towards the binding site and therefore provides ligands with an opportunity for hydrogen bond formation. It is noted that epibatidine is not involved in polar interactions both with Q114 or the indole nitrogen of W53 in the chimeric α7/Ls-AChBP protein structure.

### 2.2 Hierarchical in silico screening on proprietary and commercial compound collection

**Figure 3:**
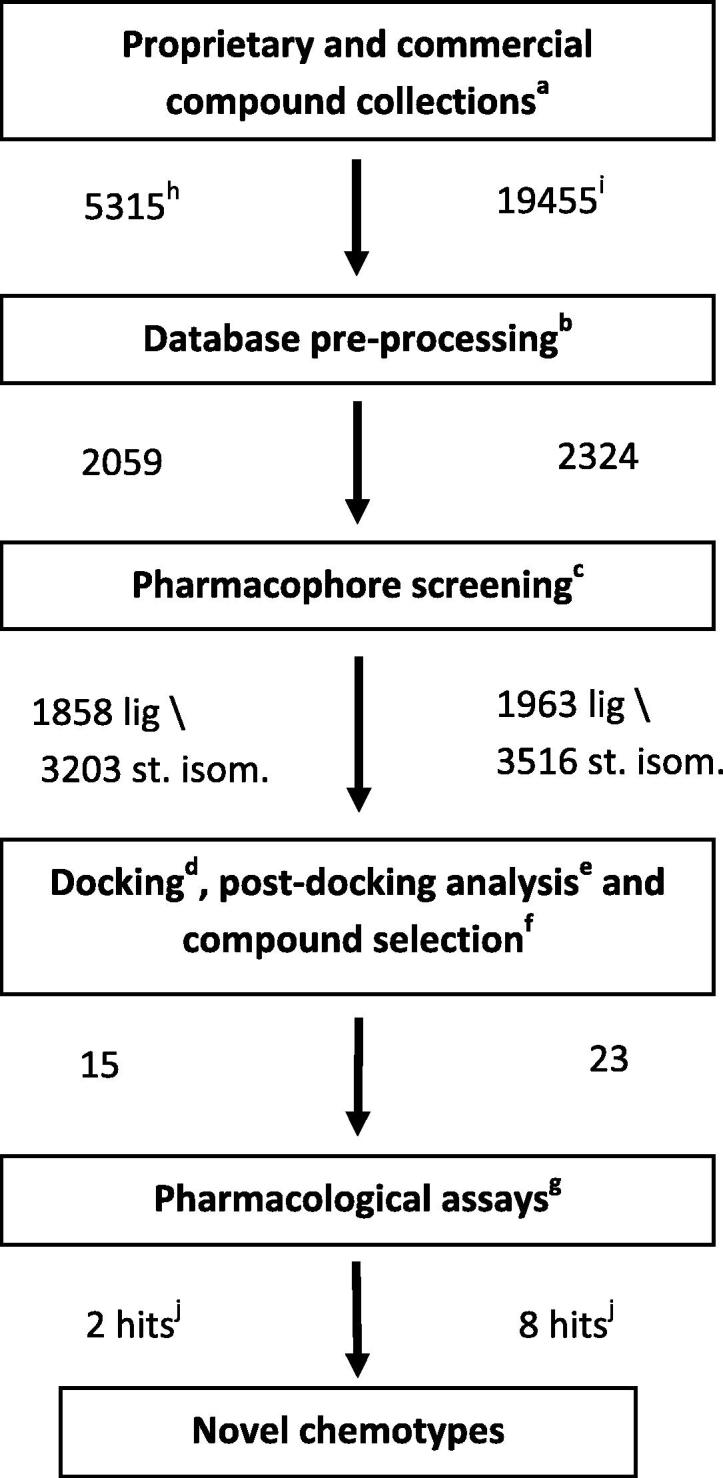
Schematic representation of our hierarchical in silico screening procedure using the crystal structure of the α7/Ls-AChBP chimeric protein (PDB: 3SQ6). (a) generation of 3D coordinates, protonation; (b) selection of compounds with cationic centers, conformation generation; (c) pocket volume, cationic center near W145; (d) generation of stereoisomers, docking; (e) ranking, cation–π interactions; (f) favorable poses, novel chemical structures; (g) binding assays; (h) proprietary database; (i) world diversity set; (j) compounds with at least p*K*_i_ ⩾5. lig: ligands; st. isom.: stereoisomers.

We have previously successfully applied a hierarchical in silico screening procedure, using a nicotine-bound Ls-AChBP cocrystal structure, to both a proprietary and commercial compound collection. In the current project, we developed and applied a similar in silico screening procedure using the cocrystal structure of the human α7/Ls-AChBP chimeric protein in complex with the agonist epibatidine. The procedure consists of several ligand selection steps with increasing complexity: (1) pre-selection of ligands containing cationic centers (quaternary or protonated nitrogen atoms), (2) a protein-based pharmacophore filter that selects ligands with the appropriate size, shape and location of the essential cationic center, (3) docking studies using the human α7/Ls-AChBP chimeric protein crystal structure, and (4) post-processing of docking poses (Fig. 3).

For comparison, we have used the same proprietary compound collection (a diverse subset of 5315 drug-like compounds) as in our previous study.^16^ All compounds with a cationic center were selected (2059 compounds) and subjected to a conformation generation procedure prior to a pharmacophore screening. Subsequently, 1858 compounds were selected using the pharmacophore screening step. Since the GOLD docking program does not generate stereoisomers during the docking procedure, a total of 3203 stereoisomers was generated (approximately 1.7 stereoisomers per compound) using the ‘chiral_enumeration’ tool available for the MOE software package (CCG, Montreal).

**Figure 4:**
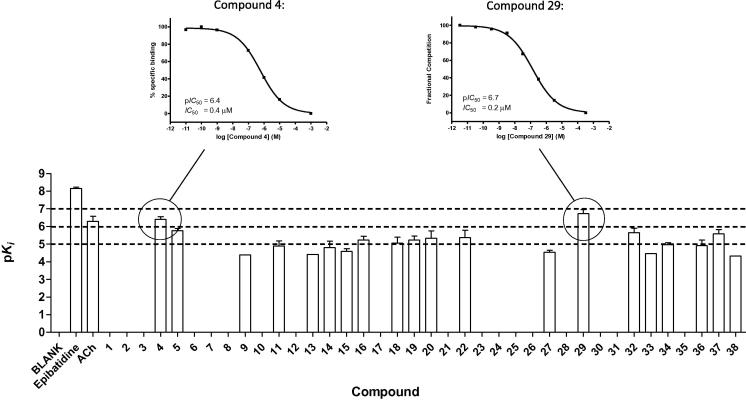
The binding affinities (p*K_i_*) for the 38 selected ligands, epibatidine (p*K*_d_) and acetylcholine have been determined from 10-point competition curves with [^3^H]epibatidine. Representative competition curves are shown for the 2 most potent ligands, compound **4** (p*K_i_* = 6.4 ± 0.2, *n* = 3) and compound **29** (p*K_i_* = 6.7 ± 0.2, *n* = 4). In the bar graph above, compounds that showed binding with 10-point curves are shown as the mean ± S.E.M., *n* ⩾3. Note that measurements of epibatidine affinity were made using the [^3^H]-radioligand and are therefore a p*K*_d_. Note that the values for compounds with low potencies (p*K_i_* <5) could be unreliable.

**Figure 5:**
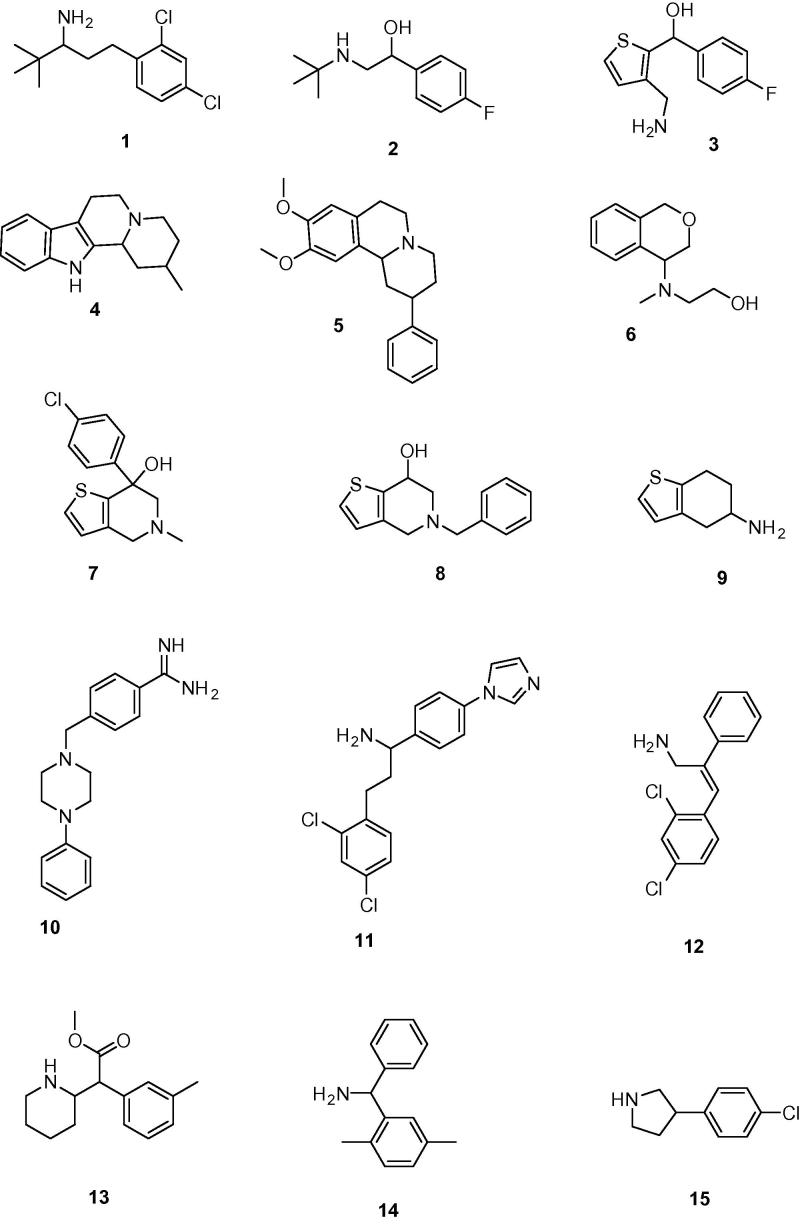
The chemical structures of the 15 ligands selected from our proprietary compound collection.

These 3203 compounds were docked into the binding pocket of the chimeric α7/Ls-AChBP protein (PDB: 3SQ6) using the GOLD Suite software package (v5.1, CCDC, Cambridge, UK). In order to select poses that are involved in cation–π interactions, deemed essential for binding, the distances between the CD2 atoms of W145 and the nitrogen atoms of the docked compounds were determined. All poses for which this distance was larger than 5.5 Å were discarded. In addition, all poses in which parts of the compounds are positioned outside the binding pocket and/or the compounds show intra- or intermolecular steric clashes were also discarded. The top-ranked 400 poses of the remaining compounds were visually inspected for steric complementarity between compound and binding pocket and for the occurrence of cation–π interactions. In addition, when evaluating the binding poses, the formation of hydrogen bonds (with the backbone carbonyl group of W145^20^ and/or with the side chain hydroxyl group of Y91) was preferred but not considered a strict requirement. Other possible hydrogen bonds between protein and compound were also considered, such as interactions with W53 and Q114 (see Section 2.1). Finally, a diverse subset of 15 structurally new compounds (compounds **1**–**15**), which were not previously tested for α7 receptor activity, was selected for binding assays on a chimeric α7/5-HT_3_ receptor (Figs. 4 and 5).^21^

**Figure 6:**
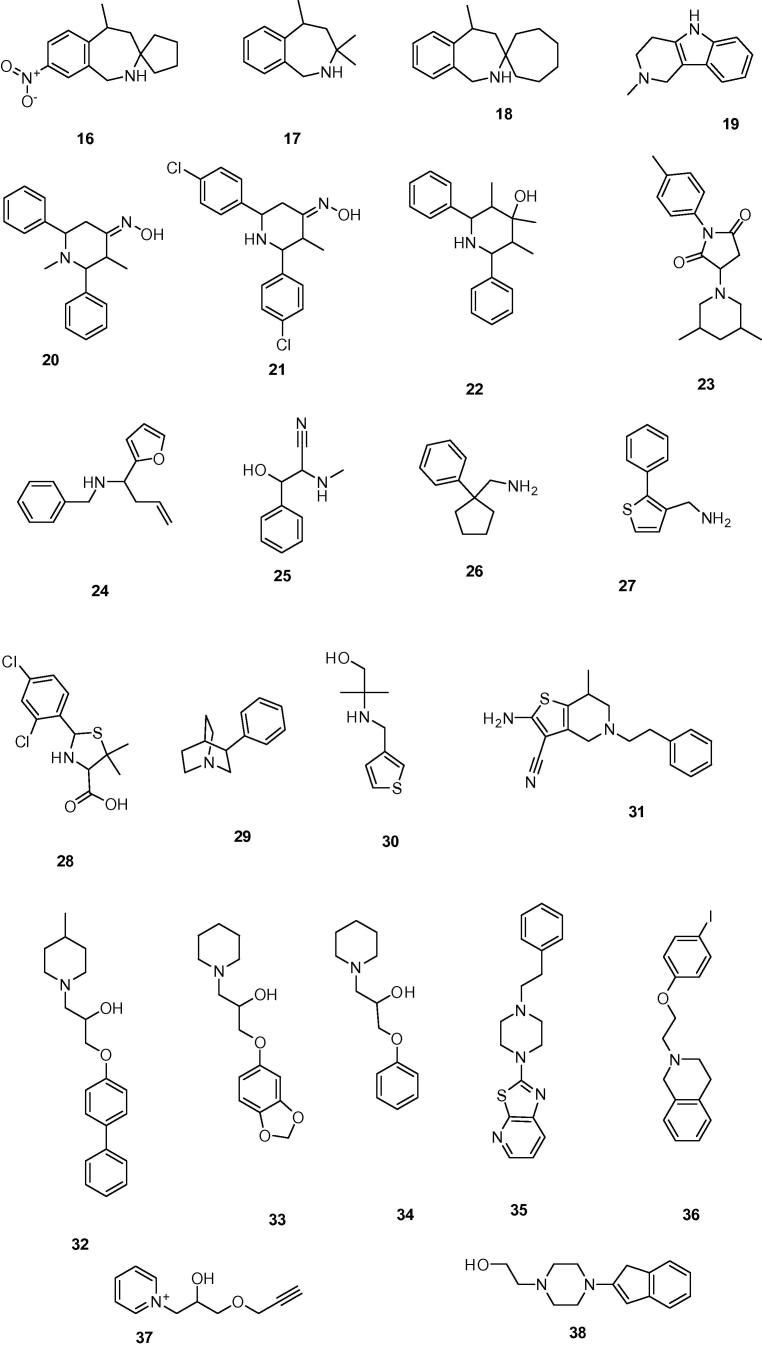
The chemical structures of the 23 ligands selected from the World Diversity Set of Specs.

Next, the World Diversity Set of Specs (WDS, obtained October 2011 from www.specs.net) was used as the commercial library. This database contains 19,455 chemically diverse compounds of which 2324 compounds contained a cationic center (quaternary or basic nitrogen atom). Similar as to the proprietary database (Fig. 3), 1963 compounds were selected using a pharmacophore screening step and 3516 stereoisomers were generated (approximately 1.8 stereoisomers per compound). These 3516 stereoisomers were docked into the chimeric α7/Ls-AChBP protein (PDB: 3SQ6) and after the post-processing step, the best-ranked 350 poses were visually inspected to yield a subset of 23 structurally new and diverse compounds for binding assays on the human α7 receptor (Figs. 4 and 6). As 4 of the 23 compounds were not available, close analogs were obtained to yield 23 compounds in total (compounds **16**–**38**).

### 2.3 Binding analysis on a chimeric α7/5-HT_3A_ receptor

All 38 selected compounds (compounds **1**–**38**), together with the endogenous ligand acetylcholine, were tested in [^3^H]epibatidine competition studies on a chimeric α7/5-HT_3A_ receptor.^21^ This chimeric receptor was used as it contains the extracellular N-terminal ligand binding domain of the human α7 receptor, binds α7 receptor ligands with a comparable affinity as the wild type α7 receptor and is known to express well in HEK293 cells.^21^

The binding data of the 38 compounds from the proprietary and commercial compound collection, epibatidine and the endogenous ligand acetylcholine are depicted in Figure 4. We identified 10 compounds with p*K*_i_ ⩾5 and their binding affinities are reported, together with the affinity of epibatidine and acetylcholine, in Table 1. Of the 10 hits, 2 compounds have similar or higher binding affinities than the endogenous ligand acetylcholine (compound **4**: p*K*_i_ = 6.4 ± 0.2; compound **29**: p*K*_i_ = 6.7 ± 0.2; acetylcholine: p*K*_i_ = 6.3 ± 0.3; Fig. 4 and Table 1).

### 2.4 Structural novelty

To investigate if the validated hits contain novel chemotypes for the α7 nAChR, a 2D topological similarity search (ECFP-4)^22^ has been performed against known α7 receptor ligands. This set consisted of 218 ligands in total and included well-known nicotinic receptor ligands (e.g., epibatidine, nicotine, acetylcholine, cytisine, PNU282987 and ARR-17779) supplemented with α7 receptor ligands from the ChEMBL database^23^ (molecular weight below 500 and *K*_i_ or *EC*_50_ ⩽10 μM). The results from this analysis are depicted in Table 1 and indicate that only one of the identified hit compounds (compound **29**) is chemically similar to any known α7 nAChR ligand (ECFP-4 Tanimoto similarity ⩾0.40,^24^ Table 1).

### 2.5 Binding modes of compounds 4 and 29

Our hierarchical in silico screening procedure resulted in two hit compounds that show higher binding affinity than acetylcholine on the chimeric α7/5-HT_3A_ receptor (compounds **4** and **29**, see Table 1). Here we present the binding poses of these two compounds which fulfill our criteria of ligand selection (i.e., (1) steric complementarity between pocket and ligand, (2) presence of cation–π interactions, and (3) preferably occurrence of hydrogen bonds).

**Figure 7:**
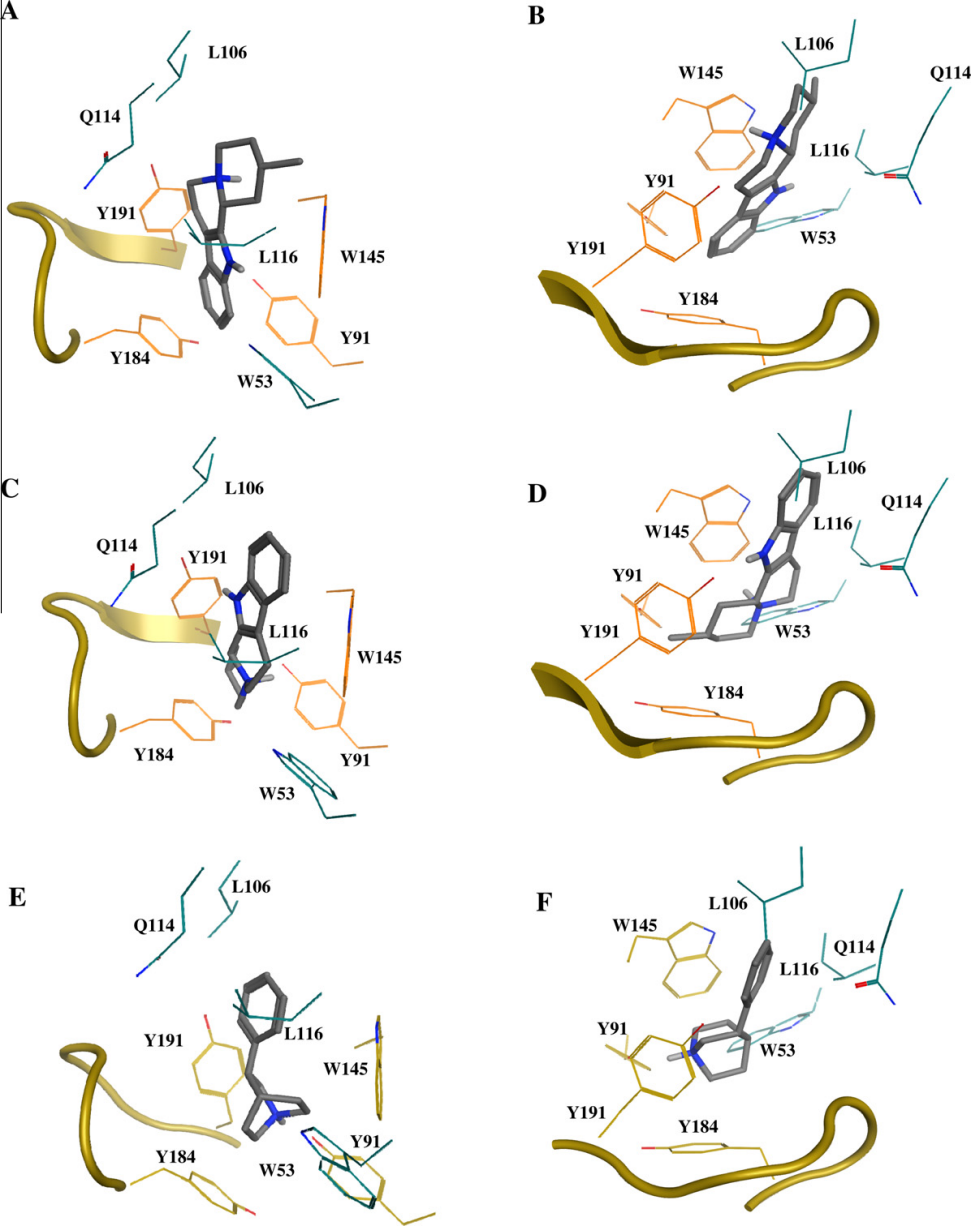
Binding poses of compounds **4** (pose 1: A, B; pose 2: C, D) and **29** (E, F) obtained after docking studies into the chimeric α7/Ls-AChBP protein crystal structure.

Two binding poses have been obtained for compound **4** in complex with the chimeric α7/Ls-AChBP protein crystal structure (Fig. 7A and B). The binding pose with the highest score (ChemScore value = 36.5449; acetylcholine ChemScore value = 19.1944; epibatidine ChemScore value = 37.5699; Fig. 7A and B) forms cation–π interactions with W145 as the cationic nitrogen atom of the ligand is at 5.4 Å distance from the aromatic side chain of W145 (atom CD2). The protonated cationic nitrogen atom is also involved in hydrogen bond formation with the carbonyl backbone of W145. Interestingly, the ligand has taken an opposite binding pose compared to epibatidine; its aromatic moiety is involved in hydrophobic contacts with the residues of the aromatic cavity within the binding pocket (W53, Y91, W145, Y184, Y191) and the basic bicyclic moiety takes a similar position as the chloropyridinyl ring of epibatidine. Hydrophobic contacts are also formed with C186 and C187 of Loop C and L106 and L116 of the complementary side.

The second binding pose of compound **4** has a lower score (ChemScore value = 34.6305; Fig. 7C and D). The cationic nitrogen atom of the ligand is located closer to the aromatic plane of W145 compared to pose 1, which could result in a stronger cation–π interaction with W145 (distance to atom CD2 of W145 = 4.9 Å). In this pose, compound **4** is also involved in extensive hydrophobic contacts with the binding site. However, compared to the first pose, the aromatic moiety of the ligand is not located in the aromatic cavity of the binding pocket and therefore not involved in π–π interactions. On the other hand, this binding pose is more similar to the binding mode of epibatidine as the aromatic and bicyclic basic parts of **4** have taken similar positions in the binding site. Furthermore, no formation of hydrogen bonds between the ligand and protein binding site is observed. It is noted that in a recent study by Srivastava et al. in which photolabeling of the human α4β2 nAChR with azidoepibatidine was combined with docking experiments in a α4β2 homology model, evidence for two distinct binding orientations of epibatine was obtained.^25^ Interestingly, the proposed binding poses of epibatidine resemble the two docking poses that we obtained for compound **4**. In the ‘up orientation’, epibatidine’s basic nitrogen atom is accommodated by the aromatic cavity whereas in the ‘down orientation’ the aromatic chloro-pyridinyl moiety is orientated toward the aromatic side chains. In addition, Brams et al*.* have shown by X-ray co-crystal structures that strychnine and d-tubucurarine adopt to different ligand orientations in the binding site of Ac-AChBP, as well.^26^

For compound **29**, only one binding pose was obtained that fulfilled our selection criteria (ChemScore value = 35.2580; Fig. 7E and F). The phenyl moiety of the ligands points towards the complementary side of the binding pocket and occupies approximately the same region as the chloropyridinyl ring of epibatidine. The cationic nitrogen atom of the ligand is buried into the aromatic cavity of the binding pocket and is close enough to W145, Y184 and Y191 to enable cation–π interactions (distances 4.9 Å, 4.7 Å and 4.6 Å, respectively). Hydrogen bonding between the cationic nitrogen atom of the ligand and the binding pocket residues are not observed in this docked pose.

## 3 Discussion and conclusions

In the current study, we have successfully applied our hierarchical in silico screening procedure on a novel crystal structure of a chimeric α7/Ls-AChBP protein. The multi-step screening protocol was applied on a proprietary compound collection and a diverse commercial compound collection. Our efforts have resulted in the identification of 10 structurally novel ligands with binding affinity for the human α7 nicotinic receptor (p*K_i_* ⩾5) and an overall hit rate of 26% was obtained (10 of 38 compounds). For the screening of the commercial compound collection an even higher hit rate of 35% (8 of 23 compounds) was observed. Two hit compounds exhibited submicromolar binding affinities (compound **4**: p*K*_i_ = 6.4 ± 0.1 and compound **29**: p*K*_i_ = 6.7 ± 0.2).

In our previous hierarchical in silico screening procedure, the crystal structure of Ls-AChBP in complex with nicotine was used and a similar procedure was applied to the same two databases as used here.^16^ In the previous study, a total of 35 compounds were selected and the binding affinity for both Ls-AChBP and the human α7 receptor was determined. Of the tested compounds, 24 had an affinity (p*K*_i_) of at least 5.0 for Ls-AChBP (∼69%) including 12 with p*K*_i_ ⩾6 (∼34%). However, for the human α7 receptor, a considerable lower hit rate was observed as only 2 compounds were identified with p*K*_i_ ⩾5 (5.0 ± 0.1 and 5.4 ± 0.1; ∼6%).^16^ In the current study the α7 hit rate was 26%, showing that the protein that shares higher homology with the actual human α7 receptor (i.e., the α7/Ls-AChBP chimeric protein) serves as a better structural template for identifying novel α7 ligands. In addition, the affinities of the hits identified with the α7/Ls-AChBP chimeric protein crystal structure are higher compared to the previous hits obtained with the Ls-AChBP crystal structure (p*K*_i_: 5.0–6.7 vs p*K*_i_: 5.0–5.4). The structural differences in residue composition and side chain orientation between the binding sites that are apparent when superposing both proteins (e.g., W53 and the tip of loop C, see superposition in Fig. 2B and D) are likely causes for the observed difference in hit rates on the α7 nicotinic receptor. For example, analyzing the docking poses of the two highest affinity hit compounds **4** and **29** in the structural superposition of the epibatidine-bound α7/Ls-AChBP chimeric protein with the nicotine-bound Ls-AChBP (Section 2.1) suggests that tight hydrophobic interactions with W53 and L116 (W53 and M114 in Ls-AChBP, respectively) are the most likely determinants for identifying them as hits in the current, but not in the previous study (Supplementary data Fig. 1). Interestingly, in our previous Ls-AChBP-based work, very close analogs of compound **4** were identified as Ls-AChBP hits but not as α7 nAChR hits (p*K*_i_ <4.5), exemplifying that subtle structural differences between compounds can have a significant effect on their selectivity profiles for homologous binding sites.

In conclusion, using a novel α7/Ls-AChBP chimeric protein crystal structure we have successfully identified novel α7 receptor ligands that may serve as chemical starting points for further optimization. In addition, the obtained results show that the novel α7/Ls-AChBP structure provides an added value when applied in structure-based virtual screening exercises that aim to identify novel ligands for the α7 nAChR.

## 4 Experimental section

### 4.1 Materials

All cell culture reagents were obtained from Gibco (Invitrogen Ltd, Paisley, UK), except fetal calf serum which was from Labtech International (Ringmer, UK). The chimeric α7/5-HT_3A_ receptor is described by Bertrand and coworkers and was cloned into pcDNA3.1 (Invitrogen, Paisley, UK) for expression in Human embryonic kidney cells (HEK293).^21^ [^3^H]epibatidine (55.8 Ci/mmol) was obtained from PerkinElmer Life Sciences (Cambridge, UK).

### 4.2 Proprietary and commercial compound collections

A proprietary compound library containing 5315 structurally diverse and drug-like compounds was used in our in silico screening procedure. The World Diversity Set was obtained from Specs (www.specs.net, October 2011) as SD files. This database contains chemically diverse screening compounds.

### 4.3 Database preparation

The compound libraries were converted into three-dimensional structures using the MOE software package (version 2010.10, Chemical Computing Group, Montreal, Canada). Counter ions and solvent molecules were filtered out, strong acids were deprotonated and strong bases were protonated, atoms were assigned with formal charges and ligands with cationic centers were selected. Subsequently, conformations of the cationic ligands were generated using a systematic search method in MOE that was adjusted for large chemical databases (conformation import function) using the default settings. No filters were set and default constraints were applied to the ligand conformation generation procedure to yield a broad range of conformations and stereoisomers. Partial atomic charges were calculated and the molecules were energy-minimized according to a steepest-descent protocol using the MMFF94x force field in MOE.

### 4.4 Pharmacophore screening

A pharmacophore screen was applied to the conformations of the cationic ligands to identify all compounds that were able to fit inside the binding pocket of the chimeric α7/Ls-AChBP protein and form cation–π interactions with W145 at the same time. To this end, the crystal structure of the chimeric α7/Ls-AChBP protein in complex with the agonist epibatidine (PDB: 3SQ6; subunits A and B) was used to construct a pharmacophore query. All heavy atoms of the residues within 7.0 Å of epibatidine were selected and used to construct an excluded volume that represents the boundaries of the binding site. We chose an atom radius of 0.8 Å for all heavy atoms. This is smaller than the atomic radius of carbon, to represent a bigger binding pocket which accounts for induced-fit effects upon ligand binding. The cationic center was defined at the position of the basic nitrogen atom of epibatidine (located at 7-azabicyclo[2.2.1]heptane moiety) with a radius of 2.5 Å. All ligands that fulfilled the requirements of this pharmacophore query were selected and subsequently their stereoisomers were generated using the chiral_enumeration SVL script of MOE. The compounds were saved as a multi-mol2 file.

### 4.5 Template preparation

The crystal structure of the chimeric α7/Ls-AChBP protein in complex with the agonist epibatidine (PDB: 3SQ6; 2.8 Å) was used in the docking procedure. The protein model was prepared using the adjacent subunits A and B. All ligand and water molecules were removed and hydrogen atoms were added using MOE. Partial atomic charges (AMBER99) were calculated and a steepest-descent energy-minimization was performed using the AMBER99 force field while keeping the heavy atoms fixed. The minimized protein structure was saved as a mol2-file.

### 4.6 Docking, post-docking analysis and selection of ligands

All selected ligands (including their stereoisomers) were docked into the orthosteric binding pocket of the chimeric α7/Ls-AChBP protein (PDB: 3SQ6) using the GOLD Suite software package (version 5.1, CCDC, Cambridge, UK) with the ChemScore scoring function and default settings, as described previously.^16^ The library screening settings were selected and the pocket for docking was assigned to be within 10 Å of the aromatic nitrogen atom (NE1) of W145. For ligands, all ring corners were allowed to flip just as the planar and pyramidal nitrogen atoms. For each ligand the three highest ranked poses were retained and the database was ranked according to the scores. Since cation–π interactions are extremely important in ligand–protein interactions with the nAChRs and AChBPs,^15,16^ we determined the distance between the approximate centroid of the W145 aromatic ring (atom CD2) and the cationic nitrogen atom of the ligand using the GOLD Suite package. The top-ranked compounds with poses that enable cation–π interactions (distance ligand cationic nitrogen to W145 centroid <5.5 Å), were visually inspected to verify optimal ligand–protein interactions. A diverse, structurally novel and high-ranked subset of ligands that are capable of forming cation–π interactions was selected for displacement studies on the human α7 receptor. The purity of all 10 experimentally validated hits was verified by liquid chromatography–mass spectroscopy (LC–MS). All hits had a purity of 96% or higher (see Supplementary data Table 2).

### 4.7 ECFP-4 2D similarity search

Two dimensional similarity searches were carried out using the ECFP-4 (extended connectivity fingerprints^22^) descriptor available in Pipeline Pilot (version 6.1.5; Accelrys, San Diego, CA). All 38 selected ligands were compared to known nicotinic receptor and α7 receptor ligands from the ChEMBL database (218 compounds; molecular weight below 500 and *K*_i_ or EC_50_ ⩽10 μM). Only the score and structure of the most similar known ligand is reported for each of the selected compounds (Table 1).

### 4.8 Cell culture

HEK293 cells were maintained on 90 mm tissue culture plates at 37 °C and 7% CO_2_ in a humidified atmosphere. They were cultured in DMEM:F12 with GlutaMAX™ I media (Dulbecco’s Modified Eagle’s Medium/Nutrient Mix F12 (1:1), Invitrogen, Paisley, UK) containing 10% fetal calf serum. For radioligand binding studies cells in 90 mm dishes were transfected using polyethyleneimine (PEI, 25 kDa, linear powder, Polysciences Inc, Philadelphia, USA). 30 μl PEI (1 mg/ml), 5 μl cDNA and 1 ml DMEM were incubated for 10 min at room temperature, added drop wise to a 70–80% confluent plate, and incubated for 2–3 days before harvesting.

### 4.9 Radioligand binding assay on chimeric α7/5-HT_3A_ receptor

Transfected HEK293 cells were harvested into 1 ml of ice-cold HEPES buffer (10 mM, pH 7.4) and frozen. After thawing, they were washed with HEPES buffer, resuspended, and 50 μg of cell membranes incubated in 0.5 ml HEPES buffer containing 3 nM [^3^H]epibatidine. Non-specific binding was determined using 3 mM −/− nicotine. For competition binding (10-point) reactions were incubated for at least 2 h at 4 °C. Reactions were terminated by vacuum filtration using a Brandel cell harvester onto GF/B filters pre-soaked in 0.3% polyethyleneimine. Radioactivity was determined by scintillation counting using a Beckman BCLS6500 (Fullerton, California, USA). Individual competition binding experiments were analyzed by iterative curve fitting using the following equation in Prism (version 4.03, GraphPad Software, Inc., San Diego, CA):

$$y=B_{\text{min}}+\frac{B_{\text{max}}-B_{\text{min}}}{1+10^{[L]-\text{log}{\text{IC}}_{50}}}$$

where *B*_min_ is the non-specific binding, *B*_max_ is the maximum specific binding, [*L*] is the concentration of competing ligand and IC_50_ is the concentration of competing ligand that blocks half of the specific bound radioligand. Values are shown for a series of experiments (*n* ⩾3) and presented as the mean ± S.E.M.

## Figures and Tables

**Table 1:** α7/5-HT_3A_ receptor binding affinities (p*K*_i_) of validated hit compounds (p*K*_i_ ⩾5) selected by a hierarchical in silico screening protocol (Fig. 3) against the chimeric α7/Ls-AChBP protein crystal structure

Compd	Structure^a^	p*K*_i_ α7/5-HT_3A_^b^^,^^c^	GOLD^d^ (rank)	ECFP-4^e^	Closest known nAChR ligand^f^	ChEMBLdb ID code
ACh		6.3 ± 0.4	—	—	—	—
Epi		8.2 ± 0.1^g^	37.5699 (62/350)	—	—	—
**4**		6.4 ± 0.1	36.5449 (83/400)	0.25		Tropisetron
**5**		5.8 ± 0.1	36.5960 (82/400)	0.30		491494
**16**		5.2 ± 0.2	35.8701 (134/350)	0.23		178291
**18**		5.1 ± 0.3	43.1003 (4/350)	0.19		523647
**19**		5.2 ± 0.2	35.5781 (151/350)	0.25		108799
**20**		5.3 ± 0.4	h	0.25		452455
**22**		5.4 ± 0.4	42.5465 (6/350)	0.22		526281
**29**		6.7 ± 0.2	35.2580 (173/350)	0.44		41294
**32**		5.7 ± 0.2	h	0.33		1739327
**37**		5.6 ± 0.2	30.0353 (300/350)	0.13		1739327

GOLD docking scores (ChemScore) and the closest structural similarity to reference nicotinic receptor ligands are given for each validated hit based on ECFP-4 2D similarity searches.

a The exact stereochemical composition of the samples tested is not known.

b p*K*_i_ values are calculated from at least three independent measurements as the mean ± S.E.M.

c Measured by displacement of [^3^H]epibatidine binding using membranes of HEK293 cells transiently expressing an α7/5-HT_3A_ chimeric receptor.

d Compounds BS7122 and BS7128 were selected from the top-ranked 400 compounds from the proprietary database. The other compounds have been selected from the top-ranked 350 compounds from WDS.

e ECFP-4 circular fingerprint Tanimoto similarity to closest known nAChR ligands in the ChEMBL database. A similarity higher than 0.40 is considered significant.^25^

f Chemical structure of the nicotinic receptor ligand in the ChEMBL database with the highest structural similarity to the validated hit compound.

g Please note that measurements of epibatidine affinity were made using the [^3^H]-radioligand and are therefore a p*K*_d_.

h These compounds have not been selected with the in silico screening, but are the closest available analogs from WDS. ACh: acetylcholine; Epi: epibatidine.
