# Supplementary material for: Identification of novel α7 nicotinic receptor ligands by in silico screening against the crystal structure of a chimeric α7 receptor ligand binding domain
Source: Bioorg Med Chem. 2012 Oct 1;20(19):5992–6002. doi: 10.1016/j.bmc.2012.06.054 (PMC3460237; doi:10.1016/j.bmc.2012.06.054)
Supplement: Supplementary data — Supplementary Figure and Tables. [file mmc1.doc]

**Identification of novel α7 nicotinic receptor ligands by in silico screening against the crystal structure of a chimeric α7 receptor ligand binding domain**

Atilla Akdemir^a,#^, Ewald Edink^b,#^, Andrew J. Thompson^c^, Sarah C.R. Lummis^c^,
Albert J. Kooistra^b^, Chris de Graaf^b^ and Iwan J.P. de Eschb,[[1]](#footnote-2)*

^a^ Division of Pharmacology, Faculty of Pharmacy, Bezmialem Vakif University, Istanbul, Turkey

^b^ Leiden/Amsterdam Center of Drug Research (LACDR), Division of Medicinal Chemistry, Amsterdam Institute for Molecules, Medicines and Systems(AIMMS), VU University Amsterdam, The Netherlands

^c^ Department of Biochemistry, University of Cambridge, Cambridge, UK

**Supplementary Information**

**
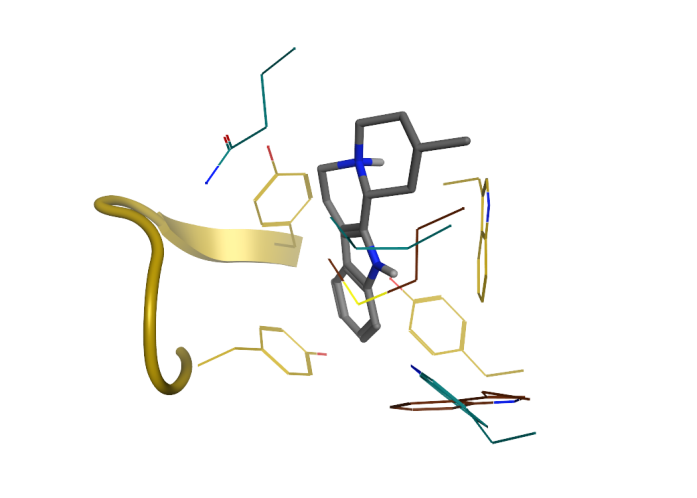

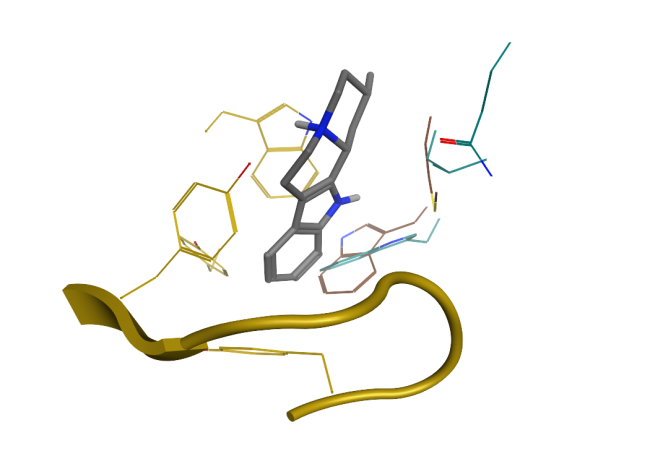
**

**Q114**

**Q114**

**W145**

**L116**

**L116**

**W145**

**Y191**

**W53**

**Y191**

**M114**

**M114**

**Y91**

**Y91**

**Y184**

**W53**

**Y184**

**
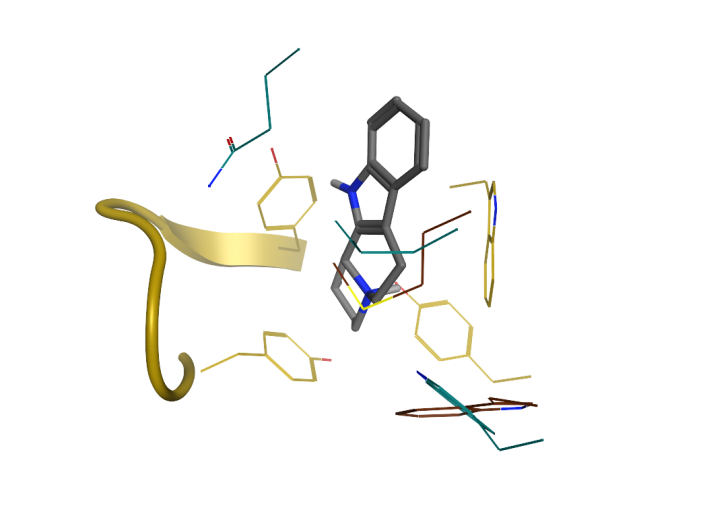

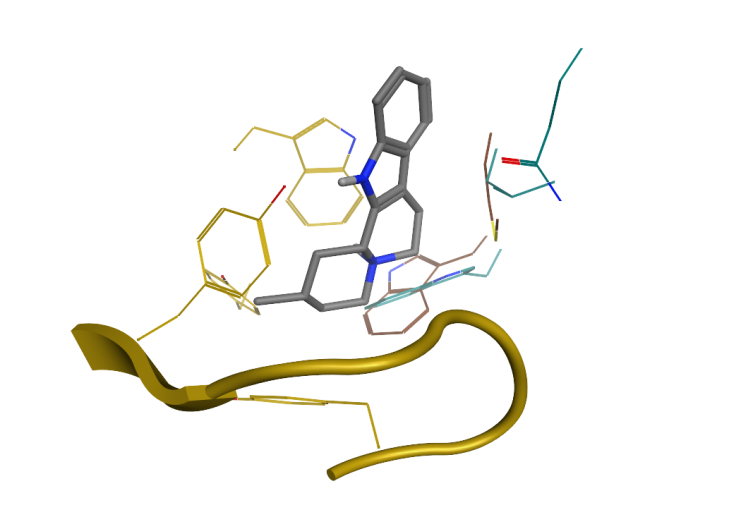
**

**Q114**

**Q114**

**W145**

**L116**

**L116**

**W145**

**Y91**

**W53**

**Y191**

**Y191**

**M114**

**M114**

**Y91**

**W53**

**Y184**

**Y184**

**
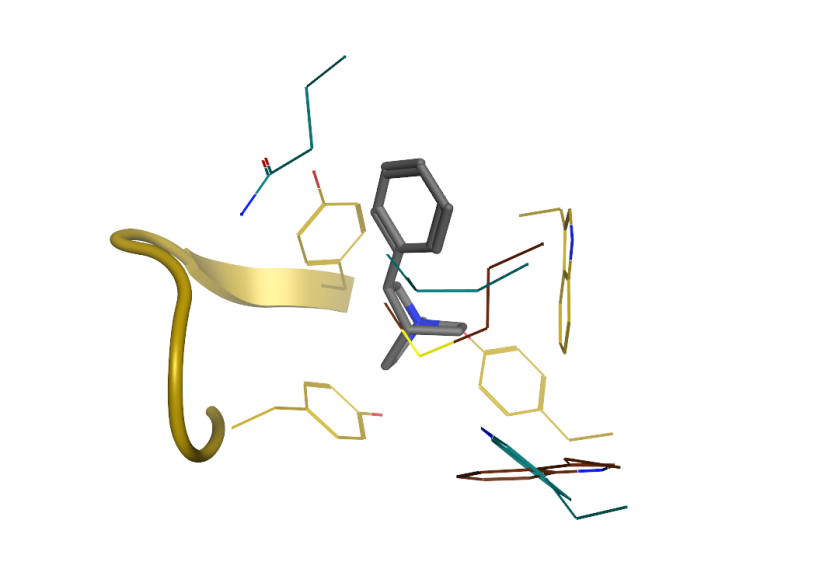

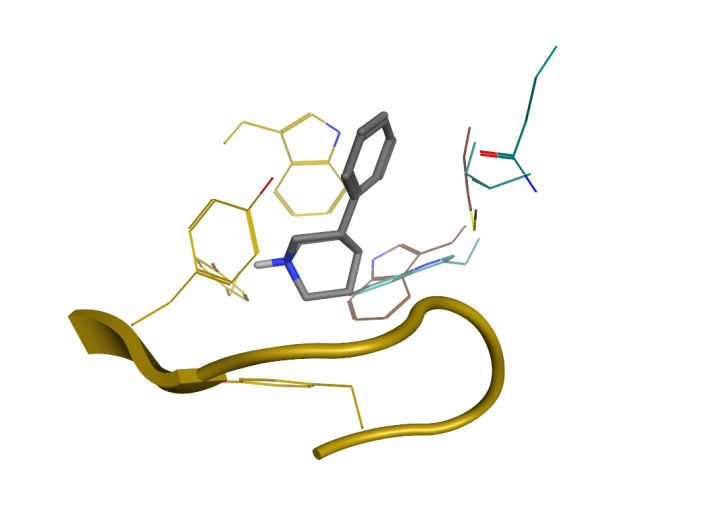
**

**Q114**

**Q114**

**W145**

**W53**

**Y191**

**M114**

**L116**

**L116**

**W53**

**Y191**

**W145**

**Y91**

**Y91**

**M114**

**Y184**

**Y184**

**Supplementary Figure 1.** The docked poses of compounds **4** (pose 1: A, B; pose 2: C, D) and **29** (E, F) in the chimeric α7/Ls-AChBP crystal structure. The crystal structure of Ls-AChBP has been superposed on the chimeric α7/Ls-AChBP protein using the Cα-atoms to reveal the differences in the binding pockets. The colouring of the chimeric α7/Ls-AChBP protein is similar to Figure 7. For clarity, only W53 and M114 of Ls-AChBP are shown (carbon atoms in brown).

**Table T1.** Supplier IDs of all compounds from the WDS obtained from Specs.

| **Compound ID** | **Supplier ID** |
| --- | --- |
| **16** (VUF 14168) | AM-760/11601007 |
| **17** (VUF 14170) | AM-760/13523007 |
| **18** (VUF 14172) | AM-760/14223002 |
| **19** (VUF 14155) | AE-473/30364041 |
| **20** (VUF 14161) | AG-690/11020061 |
| **21** (VUF 14169) | AM-760/12721006 |
| **22** (VUF 14171) | AM-760/13743005 |
| **23** (VUF 14175) | AN-919/13592024 |
| **24** (VUF 14162) | AG-690/36873062 |
| **25** (VUF 14167) | AK-823/41252461 |
| **26** (VUF 14165) | AJ-292/09732029 |
| **27** (VUF 14154) | AA-516/33241013 |
| **28** (VUF 14164) | AJ-264/34033010 |
| **29** (VUF 14160) | AG-680/20240019 |
| **30** (VUF 14174) | AN-465/43369999 |
| **31** (VUF 14173) | AN-465/14527004 |
| **32** (VUF 14176) | AP-124/41430783 |
| **33** (VUF14158) | AF-399/13374021 |
| **34** (VUF14156) | AE-641/03239022 |
| **35** (VUF14163) | AI-204/31680036 |
| **36** (VUF14166) | AJ-292/42546092 |
| **37** (VUF14157) | AE-641/30407008 |
| **38** (VUF14159) | AG-401/13980004 |

**Table T2.** Purity data for each of the validated compounds as measured by LC-MS^a^.

| **Compound** | **Retention time (min)** | **Λ (nm)** | **Purity** | **Detected mass**  **[M+H]** |
| --- | --- | --- | --- | --- |
| **4** (BS7128) | 2.84 | 230 | >99% | 241.00 |
| **5** (BS7122) | 4.13 | 230 | >99% | 324.00 |
| **16** (VUF14168) | 3.02 | 230 | 97% | 261.00 |
| **18 (**VUF14172) | 3.76 | 200 | 96% | 244.05 |
| **19** (VUF14155) | 2.39 | 230 | >99% | 186.95 |
| **20** (VUF14161) | 3.36 | 230 | >99% | 295.00 |
| **22** (VUF14171) | 3.10 | 200 | >99% | 296.00 |
| **29 (**VUF14160) | 2.65 | 230 | 97% | 187.95 |
| **32 (**VUF14176) | 3.74 | 230 | >99% | 326.00 |
| **37** (VUF14157) | 1.12 | 230 | >99% | 191.90 [M+] |

^a^ Analytical HPLC-MS analyses were conducted using a Shimadzu LC-20AD liquid chromatograph pump Analytical HPLC-MS analyses were conducted using a Shimadzu LC-20AD liquid chromatograph pump system with a Shimadzu SPD-M20A diode array detector. MS detection was performed with a Shimadzu LCMS-2010 EV liquid chromatograph mass spectrometer. The analyses were performed using the following conditions; Xbridge (C18) 5 μm column (50 mm × 4.6 mm) with solvent A (acetonitrile with 0.1% formic acid) and B (water with 0.1% formic acid), flow rate of 1.0 mL/min, start 5% A, linear gradient to 90% A in 4.5 min, then 1.5 min at 90% A, then a linear gradient to 5% A in 0.5 min, then 1.5 min at 5% A, total run time of 8.0 min. Compound purities were calculated as the percentage peak area of the analyzed compound by UV detection at the indicated wavelength.

1.  Corresponding author. Tel.: +31 20 59 87841; fax: +31 20 5987610; e-mail: [i.de.esch@vu.nl](mailto:i.de.esch@vu.nl)

   # These authors contributed equally. [↑](#footnote-ref-2)
